# Anti-Early Stage of Bacterial Recolonization Effect of *Curcuma longa* Extract as Photodynamic Adjunctive Treatment

**DOI:** 10.1155/2020/8823708

**Published:** 2020-12-18

**Authors:** Doosadee Hormdee, Weena Rinsathorn, Subin Puasiri, Paiboon Jitprasertwong

**Affiliations:** ^1^Division of Periodontology, Department of Oral Medical Science, Faculty of Dentistry and Research Group of Chronic Inflammatory Oral Diseases and Systemic Diseases Associated with Oral Health, Khon Kaen University, Khon Kaen, Thailand; ^2^Division of Periodontology, Department of Oral Medical Science, Faculty of Dentistry, Khon Kaen University, Khon Kaen 40002, Thailand; ^3^Division of Community Dentistry, Department of Oral Prevention Faculty of Dentistry, Khon Kaen University, Khon Kaen 40002, Thailand; ^4^Institute of Dentistry, Suranaree University of Technology, Nakhon Ratchasima, Thailand

## Abstract

**Objective:**

To evaluate the amount of *Fusobacterium nucleatum* (*F. nucleatum*) and *Prevotella intermedia* (*P. intermedia*) on subgingival recolonized plaque after mechanical debridement and photodynamic treatment by using blue light-emitting diodes (LEDs) in combination with topical *Curcuma longa* gel extract.

**Methods:**

A total of 12 subjects with stage III grade B periodontitis were recruited for the study. Maxillary posterior teeth with periodontal pocket >4 mm were selected. These teeth were examined for periodontal clinical data at baseline and at 1, 2, 4, and 6 weeks after treatment. All remaining teeth were treated by scaling and root planing (SRP). Then, the teeth were bilaterally divided using randomized split-mouth design with and without photodynamic adjunctive therapy (PDT). Samples of the subgingival microbiota were obtained in each visit. All samples were analyzed by multicolor TaqMan real-time polymerase chain reaction (PCR) for the presence of target bacteria.

**Results:**

Throughout the six-week follow-up, long-term improvement of probing depth and bleeding on probing was revealed on the PDT group. The number of subgingival *F. nucleatum* and *P. intermedia* also significantly reduced, compared to the baseline. There was a statistically significant recolonization in *F. nucleatum* and *P. intermedia* number after 2 and 4 weeks of conventional SRP, respectively. Our quantitative PCR method showed no significant recolonization of those subgingival bacteria on PDT sites throughout the 6-week study duration.

**Conclusion:**

The results showed that adjunctive photodynamic treatment by using blue LEDs in combination with topical *Curcuma longa* gel extract was effective to alter the recolonization patterns of *F. nucleatum* and *P. intermedia* after conventional debridement.

## 1. Introduction

Chronic periodontitis represents one of the most common bacterial infections and inflammatory diseases. Multibacterial colonization on supra- and subgingival tooth surfaces causes chronic destruction of the oral connective tissue and alveolar bone, which may result in tooth loss. The prevalence of several species of subgingival microorganisms has regularly been associated with periodontal diseases [1–4]. In dental plaque biofilm formation, *Fusobacterium nucleatum* (*F. nucleatum*) adhere to pellicles and early colonizers, while late colonizers, including *Prevotella intermedia* (*P. intermedia*), adhere to the outer surface of those microorganisms. While *F*. *nucleatum* is commonly detected in gingivitis and chronic periodontitis [5, 6], *P. intermedia* is detected in high frequency in lesions of chronic periodontitis, aggressive periodontitis, and acute necrotizing ulcerative gingivitis [5, 7–11]. Moreover, the presence of *F. nucleatum* in subgingival biofilm plays a significant role in coaggregation with other periodontal bacteria recolonization during the development of dental biofilms after eradication [12–14]. Additionally, some medical conditions such as pregnancy and poor glycemic control are associated with increased levels and frequencies of *F. nucleatum* and *P. intermedia* in sites with probing depth <5 mm [15, 16].

Mechanical debridement by scaling and root planing (SRP) is an essential method in the treatment of periodontal diseases by removing dental biofilm, bacterial products, and calculus [17]. However, the efficacy of SRP can be limited in cases with limited access to deep pockets, root anatomies, and tooth malalignment [18]. Thus, different adjunctive treatments, including local antiseptic and antibiotic, have been proposed to improve the clinical outcome of SRP or to reduce the numbers of pathogenic bacteria in those areas [19].

To avoid any unpleasant overdose or drug-resistant bacteria, our previous in vivo study considered the antibacterial effects of *Curcuma longa* extract against periodontal pathogen and its use as a photosensitizer in the gingival sulcus for additional photodynamic therapy (PDT) [20]. Thus, the objective of this study was to investigate the efficacy of subgingival application of 25 *μ*g/mg *Curcuma longa* gel extract along with irradiation with blue LED light (energy density = 16.8 J/cm^2^) for 2 minutes as an adjunct to conventional scaling and root planing procedures.

## 2. Materials and Methods

### 2.1. Study Design and Participants

This randomized split-mouth controlled trial was approved by the University Center for Ethics in Human Research (HE-611065). Twelve patients with moderate chronic periodontitis (periodontitis stage II and stage III) were enrolled in this study after the study purpose was explained and their written consents were obtained. The inclusion criteria were ages between 35 and 65 years old and presence of clinically identical parameter on 2 posterior teeth with a probing pocket depth (PPD) >4 mm on the first and second quadrants. Patients with systemic diseases that could influence the outcomes, pregnancy, smoking, diabetes mellitus, periodontal treatment within the last six months, and systemic antibiotic therapy within the last three months were excluded.

### 2.2. Clinical Examination and Treatments

The following clinical parameters were assessed at baseline and 1, 2, 4, and 6 weeks after the treatment: plaque index (PI) [21], periodontal pocket depth (PPD), clinical attachment level (CAL), and bleeding on probing (BOP) [22]. Six sites per tooth on maxillary posterior teeth were measured and recorded by the same periodontist. The PI was expressed as scores of 0 to 3. PPD and CAL were recorded as the distances from the bottom of the pocket to the gingival margin and cementoenamel junction, respectively. BOP was recorded as “present” or “absent” within 30 seconds of probing. The treatment started by giving instructions on oral hygiene and full-mouth SRP with periodontal curettes and ultrasonic devices. One randomly chosen (by coin toss) clinically identical posterior teeth also received PDT following SRP. In the PDT group, *Curcuma longa* gel extract at a concentration of 25 *μ*g/mg was applied to the gingival sulcus. Then, the chosen tooth was immediately irradiated with a 420–480 nm wavelength blue LED light source with a power output of 1000–1200 mW/cm^2^. The blue LED was connected to a medical-grade quartz, periodontal probe-like shape, round end point with a diameter of 1 mm (energy density = 16.8 J/cm^2^). The tip was lightly run along the tooth surface on both buccal and palatal sides for 2 minutes without removing it from the gingival sulcus.

### 2.3. Microbial Samples

Subgingival plaque samples were collected from the clinically identical sites on quadrants 1 and 2 at the baseline and 1-, 2-, 4-, and 6-week follow-up visits by the same periodontist and then coded by a blinded assistant. In brief, the supragingival biofilm was removed by sterile cotton pellets; each site was dried and isolated from the saliva with sterile cotton rolls; 3 sterile paper points were inserted into the periodontal pocket to collect the subgingival biofilm and were immediately suspended in 0.5 mL of RNAlater storage solution (Sigma-Aldrich, St. Louis, United States) and stored at −20°C. In the next stage, DNA was isolated from the samples using commercial kits for DNA isolation, GeneJET (Fermentas). Quantification of *F. nucleatum* and *P. intermedia* was performed by using multicolored real-time polymerase chain reaction, TaqMan amplification, and ABI FAST 7500 sequence detection system. The primers, probes, and amplification conditions are shown in Table 1 [23]. *F .nucleatum* ATCC 25586 and *P. intermedia* ATCC 25611 were used as the standard references.

### 2.4. Statistical Analysis

Statistical analysis was performed using SPSS ver. 19.0 (IBM Co., Armonk, NY, USA). The clinical parameters were calculated as means for each group and for each time point, before, and after the treatment protocols. Comparison of the baseline values was performed using the paired *T*-test. To assess the effects of the PDT on bacteria recolonization in the same periodontal pocket, the Friedman test and Wilcoxon signed-rank test were carried out. The level of significance was set at *p* < 0.05.

## 3. Result

### 3.1. Clinical Outcomes

A total of 12 patients were included in the study, including 6 male and 6 female patients, with a mean age of 53.27 ± 7.47 years. No significant differences (*p* < 0.05) were identified in the evaluated clinical parameters (PD, CAL, PI, and BOP) between the two study groups at baseline. The intergroup comparison showed no statistical significance in any of the follow-up periods throughout the study (*p* < 0.05). In the PDT group, the mean difference in the reduction of PD and CAL was statistically significant in the intragroup comparison from the 1^st^ week up to 4^th^ week of follow-up. On the other hand, only a week after conventional treatment, a significant reduction in PD was observed. Analysis of BOP in PDT revealed a statistically significant reduction from baseline to 6 weeks, but in the conventional treatment, this reduction was significant only up to 4 weeks compared to the baseline. Clinical outcomes at baseline and 1, 2, 4, and 6 weeks after treatment are presented in Table 2.

### 3.2. Bacterial Profile

**Figure 1:**
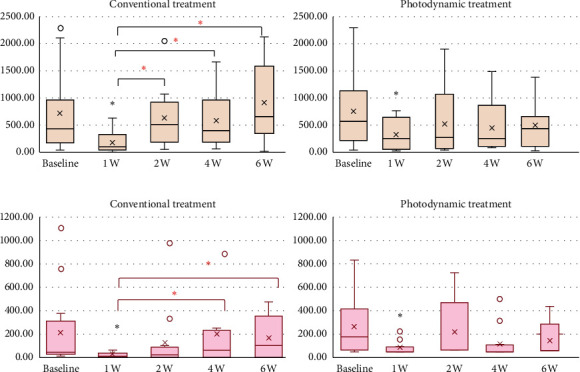
The median, mean, 25th to 75th percentiles, maximum, minimum, and maximum outlier of (a) *F. nucleatum* and (b) *P. intermedia* from baseline through 6 weeks of follow-up.

**Figure 2:**
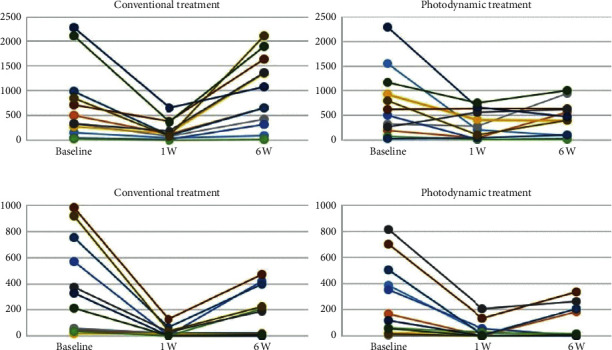
Effect of conventional and photodynamic treatments on the amount of *F. nucleatum* copy unit (a) and *P. intermedia* copy unit (b) from each 12 chronic periodontitis patients at baseline, a week, and 6 weeks following nonsurgical periodontal treatment.

Microbiological data are shown in Table 3. All groups showed a significant reduction in the amount of *F. nucleatum* and *P. intermedia* after 1 week of treatment (*p* < 0.02). When compared to 1 week after treatment, the conventional treatment group showed significantly lower reducing effects of *F. nucleatum* (*p* < 0.02) and *P. intermedia* (*p*=0.03) from the 2 and 4 weeks after treatment, respectively. In contrast, there was no statistically significant increase in the amount of *F. nucleatum* and *P. intermedia* after treatment with PD throughout the entire study period. The median, mean, 25th to 75th percentiles, maximum, minimum, and maximum outlier of *F. nucleatum* and *P. intermedia* from baseline through 6 weeks of follow-up are presented in Figure 1. Effect of conventional and photodynamic treatments on the amount of *F. nucleatum* copy unit and *P. intermedia* copy unit from each site of all 12 chronic periodontitis patients at baseline, a week, and 6 weeks following nonsurgical periodontal treatment is shown in Figure 2.

## 4. Discussion

Several studies have shown that access limitations during scaling root planing and unfavorable clinical response to conventional treatment appeared to be a consequence of subgingival ecological changes associated with increased gingival inflammation and clinical attachment loss [15, 24, 25]. In the present clinical study, periodontal examinations showed improvement in the inflammatory reactions, and there was a >10% reduction in pocket depth following one complete SRP. While these reductions gradually rebound back, we observed continual beneficial effects of *Curcuma longa* extract-mediated photodynamic therapy for up to 6 weeks.

In a classical study, recolonization of the subgingival microflora after 7 days of SRP was similar to that of periodontal healthy sites [26]. Even though cultural and dark‐field examination data have detected a nonspecific recolonization of bacteria at the 21‐day sampling point, our TaqMan quantitative DNA examination revealed a significant reemergence of subgingival *F. nucleatum* and *P. intermedia* after 14 and 21 days of the conventional SRP. Interestingly, this gradual accumulation of two target subgingival bacteria was found only in conventional treatment but not in the photodynamic therapy group. Our findings are in accordance with the previous photodynamic therapy study which reported antiperiopathogenic bacteria and *F. nucleatum* from the gingival sulcus [27]. However, the previous report was limited to only young adults with mild to moderate gingivitis.

In recent decades, many reports have proposed different wavelengths and light energies against biofilm growth in vitro [28–30]. In our previous study, we have established the optimal concentration of *Curcuma longa* extract solution and the use of blue light energy as an effective photosensitizer in PDT against periodontal bacteria [20]. It was also noted that the same dose of photosensitizer in gel form and blue light from periodontal probe-like quartz (subgingival 25 *μ*g/mg *Curcuma longa* extract irradiated with blue LED light energy density = 16.8 J/cm^2^) can be used to attain significant therapeutic effects without any local and systemic adverse effects in mild to moderate chronic periodontitis patients with healthy medical condition.

We conclude that *Curcuma longa* gel extract and blue LEDs can be used in the gingival sulcus for photodynamic adjunctive therapy to suppress subgingival recolonization of *F. nucleatum* and *P. intermedia* following conventional mechanical debridement with no detectable damage to the adjacent areas.

Wilcoxon signed-rank test was used to compare within-group differences (^#^*p* < 0.05, compared with baseline; ^*∗*^*p* < 0.05, compared with 1 week after treatment).

The median (solid bar), mean (cross), 25th to 75th percentiles (box), maximum and minimum (whisker), and maximum outlier (dot) were indicated. The black and red asterisks indicate a statistically significant difference of bacterial unit when compared with baseline and the first week of follow-up, respectively (*p* < 0.05).

## Figures and Tables

**Table 1:** Target primer/probe sequence and amplification condition for quantitative real-time PCR used in this study.

	Primer sequence (5′-3′)	Thermal program
*Prevotella intermedia*	F : CCACATATGGCATCTGACGTG	10 min at 95°C, 40 cycles at 95°C for 15 s, 58°C for 60 s, 72°C for 45 s, and a final extension at 72°C for 5 min
	R : TCAATCTGCACGCTACTTGG	
	P : **FAM**-ACCAAAGATTCTACGGTGGAGGATGGG-**QSY**	
*Fusobacterium nucleatum*	F : CGCAGAAGGTGAAAGTCCTGTAT	
	R : TGGTCCTCACTGATTCACACAGA	
	P : **ABY**- ACTTTGCTCCCAAGTAACATGGAACAC GAG-**QSY**	

F: forward primer; R: reverse primer; P: probe; FAM: 6-carboxyfluorescein.

**Table 2:** Mean clinical data from baseline through 6 weeks of follow-up of the study groups.

Clinical parameters	Conventional treatment					PDT				
	Baseline	After treatment (weeks)				Baseline	After treatment (weeks)			
		1	2	4	6		1	2	4	6
Pocket depth (mm)	4.38 ± 0.61	3.88 ± 0.41^*∗*^	3.96 ± 0.52	4.08 ± 0.55	4.08 ± 0.55	4.46 ± 0.68	4.00 ± 0.63^*∗*^	4.00 ± 0.79^*∗*^	4.17 ± 0.56^*∗*^	4.29 ± 0.57
Clinical attachment loss (mm)	4.00 ± 1.08	3.46 ± 1.24	3.58 ± 1.08	3.75 ± 1.09	3.83 ± 0.98	4.38 ± 1.42	3.75 ± 1.53^*∗*^	3.92 ± 1.48^*∗*^	4.04 ± 1.42^*∗*^	4.17 ± 1.38
Plaque index	1.04 ± 0.33	0.79 ± 0.16	0.79 ± 0.20	0.92 ± 0.29	0.96 ± 0.36	1.04 ± 0.28	0.85 ± 0.18	0.75 ± 0.22^*∗*^	0.81 ± 0.21	0.88 ± 0.29
Bleeding on probing (%)	85.42 ± 20.58	56.25 ± 10.54^*∗*^	58.33 ± 16.87^*∗*^	58.33 ± 12.08^*∗*^	68.75 ± 16.87	85.42 ± 20.83	54.17 ± 15.02^*∗*^	58.33 ± 11.02^*∗*^	60.42 ± 12.50^*∗*^	66.67 ± 13.18^*∗*^

Repeated ANOVA was used to compare within-group differences (^*∗*^*p* < 0.05). All data are presented in mean ± SD.

**Table 3:** Median and interquartile range of bacterial unit from baseline through 6 weeks of follow-up of the study groups.

		Baseline	After treatment (weeks)			
			1	2	4	6
*F. nucleatum*	Conventional treatment	420.94 (181–955)	102.75 (54–314)^#^	513.46 (197–895)^*∗*^	394.63 (191–953)^*∗*^	655.56 (346–1568)^*∗*^
	PDT	561.54 (215–1109)	243.65 (44–616)^#^	254.22 (70–1053)	238.14 (90–832)	433.10 (91–631)

*P. intermedia*	Conventional treatment	44.10 (28.9–302)	14.03 (0.2–35)^#^	20.40 (0.3–87)	63.55 (0.2–228)^*∗*^	104.73 (0.3–355)^*∗*^
	PDT	141.17 (30.85–378)	13.34 (0.1–52)^#^	28.21 (0.1–436)	13.08 (0.1–67)	15.79 (0.2–252)

All data are presented in median (quartile 1–quartile 3).

## Data Availability

The *Curcuma longa* gel extraction data used to support the findings of this study were supplied by Center for Research and Development of Herbal Health Products under license and so cannot be made freely available. Requests for access to these data should be made to Associate Professor Doosadee Hormdee, nootdoosadee@hotmail.com or hdoosa@kku.ac.th.
